# Comparison of Atlantic salmon individuals with different outcomes of cardiomyopathy syndrome (CMS)

**DOI:** 10.1186/1471-2164-13-205

**Published:** 2012-05-30

**Authors:** Gerrit Timmerhaus, Aleksei Krasnov, Harald Takle, Sergey Afanasyev, Pål Nilsen, Marit Rode, Sven Martin Jørgensen

**Affiliations:** 1 Nofima AS, P. O. Box 5010, N-1432, Ås, Norway; 2 Department of Animal and Aquacultural Sciences, Norwegian University of Life Sciences, P. O. Box 5003, N-1432, Ås, Norway; 3 Sechenov Institute of Evolutionary Physiology and Biochemistry, Peterburg, Russia; 4 PHARMAQ AS, P. O. Box 267, N-0213, Oslo, Norway; 5 AVS Chile SA, Puerto Varas, Chile

## Abstract

**Background:**

Cardiomyopathy syndrome (CMS) is a severe disease of Atlantic salmon (*Salmo salar* L.) associated with significant economic losses in the aquaculture industry. CMS is diagnosed with a severe inflammation and degradation of myocardial tissue caused by a double-stranded RNA virus named piscine myocarditis virus (PMCV), with structural similarities to the *Totiviridae* family. In the present study we characterized individual host responses and genomic determinants of different disease outcomes.

**Results:**

From time course studies of experimentally infected Atlantic salmon post-smolts, fish exhibited different outcomes of infection and disease. High responder (HR) fish were characterized with sustained and increased viral load and pathology in heart tissue. Low responder (LR) fish showed declining viral load from 6–10 weeks post infection (wpi) and absence of pathology. Global gene expression (SIQ2.0 oligonucleotide microarray) in HR and LR hearts during infection was compared, in order to characterize differences in the host response and to identify genes with expression patterns that could explain or predict the different outcomes of disease. Virus-responsive genes involved in early antiviral and innate immune responses were upregulated equally in LR and HR at the first stage (2–4 wpi), reflecting the initial increase in virus replication. Repression of heart muscle development was identified by gene ontology enrichment analyses, indicating the early onset of pathology. By six weeks both responder groups had comparable viral load, while increased pathology was observed in HR fish. This was reflected by induced expression of genes implicated in apoptosis and cell death mechanisms, presumably related to lymphocyte regulation and survival. In contrast, LR fish showed earlier activation of NK cell-mediated cytotoxicity and NOD-like receptor signaling pathways. At the late stage of infection, increased pathology and viral load in HR was accompanied by a broad activation of genes involved in adaptive immunity and particularly T cell responses, probably reflecting the increased infiltration and homing of virus-specific T cells to the infected heart. This was in sharp contrast to LR fish, where recovery and reduced viral load was associated with a significantly reduced transcription of adaptive immunity genes and activation of genes involved in energy metabolism.

**Conclusions:**

In contrast to LR, a stronger and sustained expression of genes involved in adaptive immune responses in heart tissue of HR at the late stage of disease probably reflected the increased lymphocyte infiltration and pathological outcome. In addition to controlled adaptive immunity and activation of genes involved in cardiac energy metabolism in LR at the late stage, recovery of this group could also be related to an earlier activation of NOD-like receptor signaling and NK cell-mediated cytotoxicity pathways.

## Background

Cardiomyopathy syndrome (CMS) is a severe cardiac disease affecting farmed Atlantic salmon (*Salmo salar* L.) primarily in the second year in seawater close to harvest [1,2]. Since the first diagnosis in Norway in 1985 [3], it has later been diagnosed in Scotland, the Faroe Island, Denmark and Canada [1,4-6]. Pathology associated with CMS has also been observed in wild Atlantic salmon [7]. CMS is diagnosed based on cardiac histopathology, characterised by a severe inflammation and necrosis of the spongy myocardium of the atrium and ventricle [4]. Inflammatory infiltrates consist of mononuclear cells, probably lymphocytes and macrophages. The compact layer of the ventricle is usually less affected, and always occurs later than changes in the spongious layer [4,8]. Farmed salmon suffering from CMS often lack clinical signs and may die suddenly due to rupture of the atrium or sinus venosus resulting in cardiac tamponade [3,4]. A remarkable feature of CMS is the slow development of pathology, which is observed both in the field and under experimental conditions [8-10].

Recently, the causative agent of CMS was identified as double-stranded RNA virus with the proposed name piscine myocarditis virus (PMCV) [9]. The same virus sequence was also identified from high-throughput sequencing of fish with CMS [11]. PMCV has a genome size of 6,688 bp with three open reading frames, the second encoding an RNA-dependent RNA polymerase showing sequence similarities with *Giardia lamblia* virus and infectious myonecrosis virus of penaeid shrimp, suggesting assignment to the *Totiviridae* family [9]. Following experimental challenge with cell culture-grown virus, histopathological changes were observed in heart tissue from 6 weeks post-infection (wpi) with peak severity at 9–10 wpi [9,10]. Analysis of viral load by quantitative real-time RT-PCR (qPCR) showed replication of virus in several organs from 4 wpi, suggesting a broad tissue tropism. Peak of viral load occurred in heart, spleen and kidney and coincided with the peak of cardiac pathology [10]. Viral load in the hearts from experimental and clinical field cases correlated well the severity of histopathological changes, suggesting that cytopathic effects of infection was a major determinant of the myocardial changes [9,10]. From transcriptome analysis of immune responses in fish, developing the strongest pathology and infection, the temporal and spatial regulation of the different arms of immunity during CMS was characterised [10]. It was shown that the peak of cardiac pathology and viral load coincided with a cardiac-specific induction of T cell response genes and splenic induction of complement genes. Activation of these responses was preceding a reduction in viral load and pathology, suggesting that they were important for viral clearance and recovery.

From the same challenge experiment, a significant proportion of the infected fish did not develop cardiac pathology, providing an opportunity for a comparative study of individuals with different outcomes of disease and characterisation of the underlying molecular mechanisms associated with protection versus pathology. Here, we use transcriptome analysis to show that fish developing sustained or increased viral load and severe pathology (so called high responders, HR) have a different character and regulation of immune responses and metabolic pathways compared to fish with viral clearance and absence of pathology (so called low responders, LR). These results provide a novel understanding of individual responses to CMS and genomic and immunological correlates of virus clearance, recovery and pathology.

## Results

### PMCV infection and disease responders

**Figure 1:**
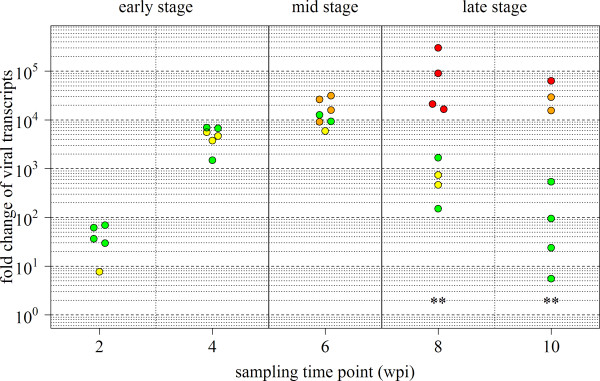
**Virus load and pathology of disease responders.** PMCV RNA level and pathology score in heart tissue of challenged fish over the time course of infection. The three stages of disease (early = 2–4 wpi, mid = 6 wpi and late = 8–10 wpi) are indicated on top of the figure, as explained in Results. Relative quantification of PMCV by qPCR is given as fold change of viral transcripts relative to the median levels of 0 wpi samples set to 1. Individual values are shown as dots and colored according to the level of CMS pathology (histopathology score level based on atrium; green = score 0, yellow = 1, orange = 2, red = 3). Significant differences in viral load between high responder fish (HR, orange and red dots) and low responder fish (LR, green and yellow dots) from mid and late stages are marked with asterisks (*t*-test on log-transformed values, p < 0.01). Overlapping dots were plotted beside each other.

From a challenge trial where fish were infected with intraperitoneal (IP) injection of identical doses of PMCV [10], we observed different outcomes of cardiac pathology between individuals. It was shown that all fish mounted a similar antiviral status (gene expression, microarrays/qPCR) and cardiac viral load (PMCV RNA, qPCR) until 6 weeks post infection (wpi). At this time point, cardiac pathology associated with CMS (histopathology score 2) was first significant and found positive in 17/27 fish. In the present study, we quantified viral load in an extended number of individuals (n = 10–15 per time point) and found that in the subsequent course of infection (8–10 wpi), fish could be divided in two groups according to differences in the development of disease (histopathology) and infection (viral load) (Figure 1). One group, termed high responders (HR), developed sustained or increased PMCV levels in heart together with elevated pathology that further decreased until 12 wpi [10]. In the other group, termed low responders (LR), viral load declined from 6–10 wpi to levels similar as for fish at 2 wpi, and no pathology was detected. According to these observations, we compared global gene expression in hearts of HR and LR during infection, in order to characterize potential differences in the host response and to identify gene expression patterns that could explain or predict the different disease outcomes.

### Overall host responses in high and low responders

**Figure 2:**
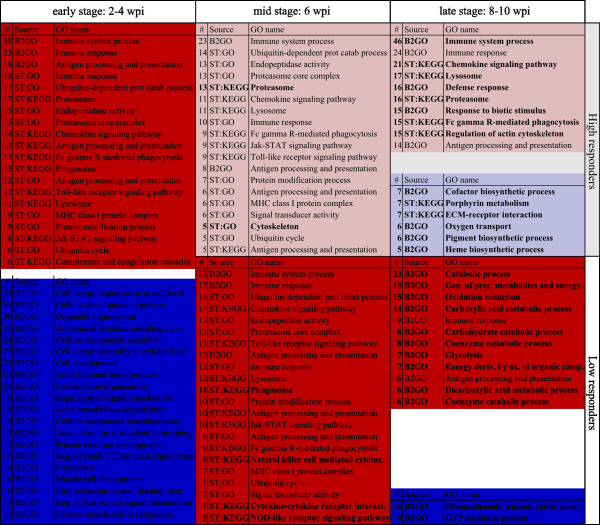
**Enrichment analysis of gene ontology classes and pathways.** Gene enrichment analysis of regulated gene ontology (GO) classes and KEGG pathways in all fish at the early disease stage, and in high (HR) and low responder (LR) groups at the mid and late stage. Categories highlighted in red indicate upregulated genes and blue indicate downregulated genes. Categories that were only regulated in one group (HR or LR) in mid and late stages are indicated with bold text. The different bioinformatics sources used for analyses are indicated (column “source”); ST:GO/KEGG: STARS platform, B2GO: BLAST2GO/Cytoscape. Column “#” shows the number of regulated genes behind the respective GO term (see Additional file 2 for primary data).

As a basis for gene expression analysis, the entire time course of disease was divided into three stages according to the characteristics of infection and pathology for HR and LR (Figure 1): the early stage (2–4 wpi) with equal viral load and histopathology score (before separation of HR/LR), the mid stage (6 wpi) with equal viral load but different histopathology score, and the late stage (8–10 wpi) where both viral load and histopathology were different between HR and LR. An overview of individual fish that were analysed from each stage is given in Additional file 1. Microarrays analysis was performed on 32 control (sham-injected) fish and 33 challenged fish, including 11 fish from the early stage and 11 HR and 11 LR fish from mid and late stages (Figure 1). For initial characterization of overall host responses in HR and LR, differentially expressed genes (DEG) were subject to enrichment analysis of gene ontology (GO) classes and KEGG pathways per each stage and responder groups (Figure 2). Common responses at the early stage included activation of innate immune responses; immune signaling pathways (Toll-like receptors, Jak-STATs, chemokines), antigen presentation (MHC class I complex) and processing (proteasome, protein modification- and ubiquitin-dependent processes), complement and phagocytosis. Repressed responses included cellular developmental processes, cytoskeleton organization and cardiac muscle development, possibly reflecting the infection-related stress and initial cellular events of pathological changes. Activation of immune responses was maintained through the mid stage for both HR and LR, with a large overlap of immune categories between the two groups (Figure 2, Additional file 2). However, three pathways were specifically activated in LR at this stage, namely cytokine-cytokine receptor interaction (7 genes), natural killer (NK) cell-mediated cytotoxicity (8 genes) and NOD-like receptor (NLR) signaling pathway (5 genes). The two latter pathways were interesting, being important for cell-mediated immune defense (NK cells) and for the innate cytosolic immune sensing and priming of antigen-specific T cell immunity (NLR pathway), yet with limited functional understanding in teleost fish. At the late stage, differences in responses between HR and LR were most prominent. While the number of upregulated immune categories increased significantly in HR, these were completely ablated or leveled off in LR. Instead, LR fish showed significant induction of different catabolic and metabolic processes, possibly reflecting responses associated with recovery.

### Gene markers and predictors of responses and disease outcome

**Figure 3:**
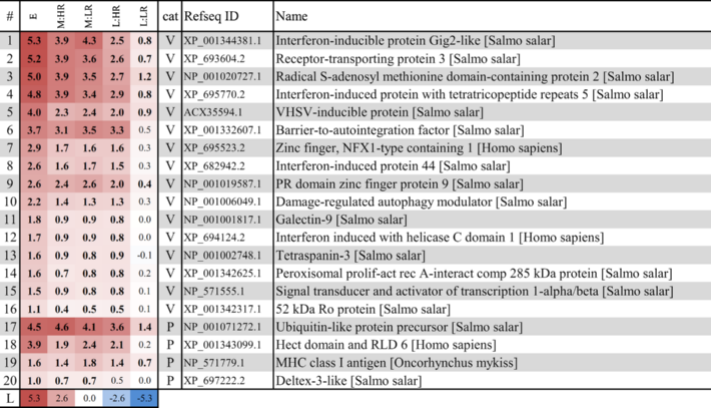
**Gene markers of early antiviral response.** Heat map of 20 selected genes with expression profile showing upregulation at the early stage and gradually decreased expression during mid and late stages of infection (complete data in Additional file 3). Left columns show the median log_2_ expression ratios of genes in the different stages and responder groups (“E” = early, “M:HR” = mid stage HR, “M:LR” = mid stage LR, “L:HR” = late stage HR, “L:LR” = late stage LR). Graded levels from white to red and blue indicate respectively upregulation and downregulation, according to the color scale (row “L”). Significant differences in log_2_–ER between control and infected samples (*t*-test, p < 0.05) are shown in bold. Column “cat” indicates the category of gene annotation according to [10]: V- early antiviral and IFN response, P- MHC antigen presentation.

**Figure 4:**
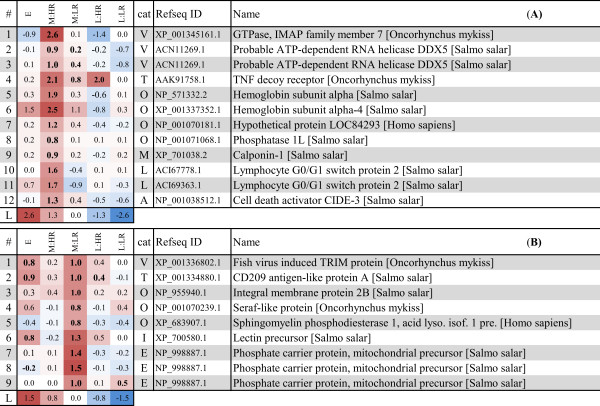
**Early pathology and outcome predictor genes.** Heat map of all genes with expression profile showing upregulation in HR (A) and LR (B) fish at the mid stage of infection. Further explanations are given in Figure 3. Column “cat” indicates the category of gene annotation according to [10]: V- early antiviral and IFN response, T- T cell response, M- muscle cytoskeleton development, L- lymphocyte regulation, A- apoptosis, I- implicated in immune response, E- mitochondrial electron chain/energy metabolism, O- other/unknown. Genes may have identical name and Refseq ID match, but represent different cDNA sequences on the array (see Additional file 3.)

**Figure 5:**
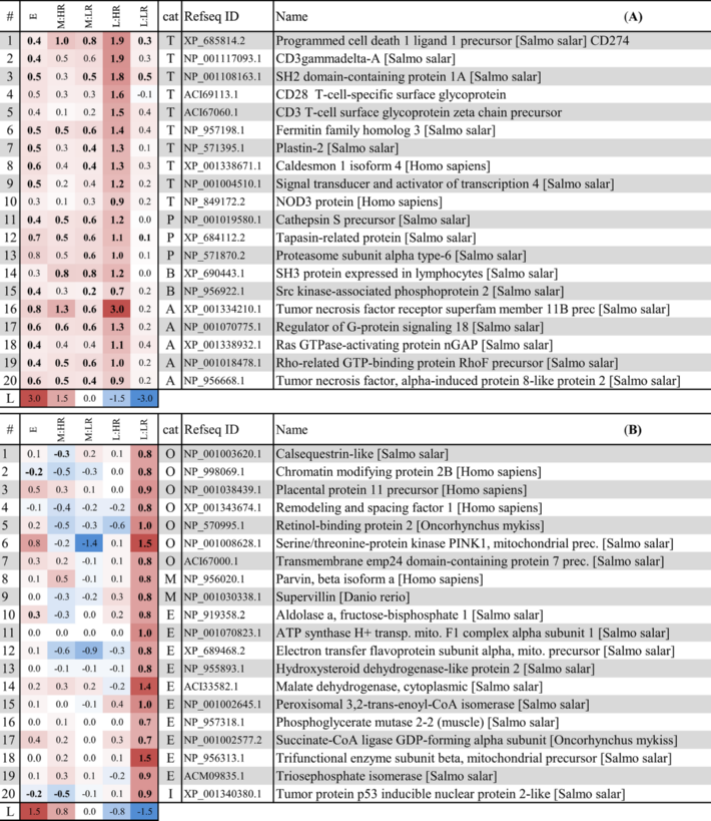
**Gene markers of pathology and outcome.** Heat map of selected genes with expression profile showing strongest upregulation in HR (A) or LR (B) fish at the late stage of infection. Further explanations are given in Figure 3. Column “cat” indicates the category of gene annotation according to [10]: T- T cell response, P- MHC antigen presentation, B- B cell response, A- apoptosis, M- muscle cytoskeleton development, E- mitochondrial electron chain/energy metabolism, I- implicated in immune response, O- other/unknown.

Further, we identified genes associated with the early response to infection and genes with expression differences between HR and LR in mid and late stages. Differentially expressed genes with contrasting expression between HR and LR (*t*-test, p < 0.05) and expression profiles that correlated to the early, mid and late stages of infection were selected (Pearson’s correlation coefficient > 0.6, Additional file 3). Heat maps of selected genes are shown in Figures 3, 4 and 5.

#### Markers of early antiviral response

We identified 250 genes that showed significant upregulation in the early stage and gradually decreased expression at the mid and late stages (Additional file 3). These genes were induced with an average log_2_-ER of +1.7 at the early stage; expression ratios of 20 genes are shown in Figure 3 (selected based on functional importance by evidence from other transcriptome studies in fish or higher vertebrates). The majority of these genes were previously identified as virus-responsive [12], and they are part of the early antiviral and interferon (IFN) response to CMS [10]. Other genes in this group encode proteins important for protein degradation and antigen presentation via MHC.

#### Early pathology and outcome predictors

Since viral clearance occurred in LR at the late stage, genes that were differentially induced or repressed in LR versus HR at the preceding mid stage might represent prognostic markers of disease outcome. Differentially expressed genes at this stage were of particular interest, since PMCV levels in heart correlated with histopathology score [10] and this was the only stage where HR and LR had different histopathology score levels but equal viral loads. Thus, such genes may represent early predictors of pathology or recovery/clearance. We identified nine genes (twelve transcripts, including one hypothetical gene) that were induced at mid stage in HR but not in LR (predictors of early pathology, Figure 4A), and seven genes (nine transcripts) that were induced in LR but not in HR (early predictors of recovery/clearance, Figure 4B). Genes induced in HR were mainly immune-related by function. *Probable ATP-dependent RNA helicase DDX5* (aka *p68*) encodes a RNA helicase regulating many aspects of transcription and shown to interact with HCV replication [13]. The other genes were implicated in apoptosis and thus might be a part of cell death mechanisms controlling lymphocyte regulation and survival. *TNF decoy receptor* (also known as TR6 or *decoy receptor 3*), is a member of the TNF receptor superfamily and an important mediator of T cell immunity and biomarker for inflammatory diseases (reviewed in [14]). Similarly, *GTPase IMAP7 family member 7* (or IAN7, GIMAP7) is part of a newly discovered family of cell survival regulators expressed in lymphocytes [15]. *Lymphocyte G0/G1 switch protein 2* (G0S2) was identified as a novel pro-apoptotic factor induced by TNF-α through NF-κB and shown to antagonize Bcl-2 [16]. Interestingly, G0S2 expression was particularly high in heart and peripheral blood cells, the latter was also observed during measles virus infection in humans [17]. Yet another HR-induced pro-apoptotic gene was *cell death activator CIDE-3* which was also specifically expressed in heart and intestine [18]. Its implication in virus infection has also been reported [19].

Genes that were induced in LR had no clear functional relation to the disease, except for *CD209 antigen-like protein A* and *lectin precursor*, presumably involved in immune-cell signaling (Figure 4B).

#### Markers of pathology and outcome

To identify genes whose expression patterns were predictive of the outcome of infection, we searched for genes that were induced during declining viral load/pathology at late stage in LR but not in HR (predictors of recovery/clearance), and genes that were upregulated during increased viral load/pathology in HR but not in LR (predictors of pathology). We identified 116 genes that were induced in HR while 33 genes were induced in LR (Additional file 3). Nearly all genes with higher expression in HR were involved in adaptive immune responses (Figure 5A), and were included in our previously identified CMS profiles for T/B cell responses, MHC class I antigen presentation and apoptosis [10]. Increased migration of leukocytic cells (e.g. macrophages and dendritic cells) was also evident from induced gene expression of several chemokines and cytokines (Additional file 3). Genes related to T cell responses were overrepresented, which likely reflected increased infiltration and homing of virus-specific T cells to the infected tissue. Upregulation of genes encoding components of the T-cell receptor (*CD3gammadelta-A, CD3 zeta chain precursor* and *CD28*) substantiated this assumption. In addition, an activator of naïve T cells, *SH2 domain-containing protein 1A* was induced. Genes with putative roles in the regulation of effector function and controlled cell death of T cells included several TNF-related genes and *programmed cell death ligand 1* (aka CD274/B7-H1). Upregulation of genes encoding Rho GTPases was also interesting, since they have been implicated in the regulation of T cell-receptor signaling and cytoskeletal reorganization, cell migration and apoptosis in T cells [20].

Genes that were induced during declining viral replication in LR and not in HR encoded for enzymes of metabolic pathways or cell respiration, and can be considered to represent markers for recovery or protection (Figure 5B). Some of these enzymes are involved in the intermediate pathways of metabolism, e.g. *aldolase atriosephosphate isomerase* and *phosphoglycerate mutase 2–2* (all involved in gluconeogenesis and glycolysis), *malate dehydrogenase* (catalytic enzyme in the citric acid cycle) and *peroxisomal 3,2-trans-enoyl-CoA isomerase* (involved in beta-oxidation of unsaturated fatty acids). Components of the electron transport chain included *ATP synthase (H + transporting mitochondrial F1 complex alpha subunit)electron transfer flavoprotein subunit alpha* and *succinate-CoA ligase GDP-forming alpha subunit*. Three genes are potentially involved in heart muscle regeneration. *Hydroxysteroid dehydrogenase-like protein 2* was induced during myocardial injury following injection of bone marrow mononuclear cells in rats [21]. *Calsequestrin-like protein* is important for the Ca^2+^ regulation in muscle cells and a dramatic decrease of protein concentration was observed in a proteomics study of human dystrophic muscle fibers [22]. *β-parvin* (aka Affixin) is a integrin-linked protein that is involved in the linkage between integrin and the cytoskeleton and was supposed to be involved in membrane repair mechanisms in human [23].

**Figure 6:**
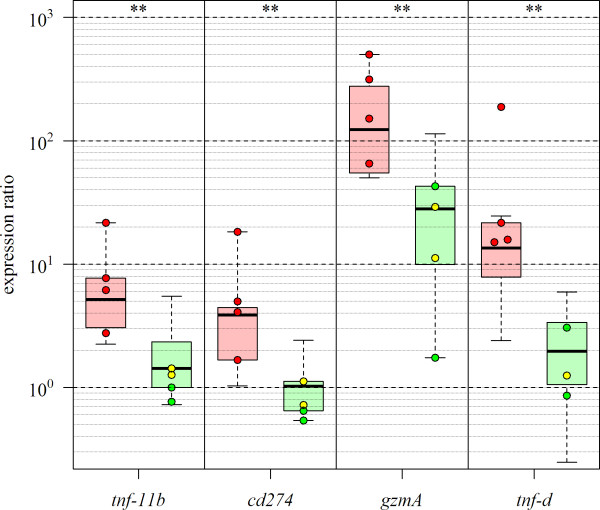
**Gene expression of selected markers of late pathology.** Real-time qPCR expression of four genes identified as pathology markers from microarrays in an extended number of individuals eight weeks post-infection. Gene abbreviations are; *tnf-11b*: *tumor necrosis factor receptor superfamily member 11b*, *cd274*: *programmed cell death ligand 1*, *gzmA*: *granzyme A*, *tnf-d*: *tumor necrosis factor decoy receptor*. The gene expression ratios are shown as boxplots based on nine HR fish (red) and ten LR fish (green) against the average of control fish. Values of the fish used in the microarray experiments are highlighted with dots and colored according to their histopathology score level (see Figure 1). Boxes represent 50% of the values, while black bars mark the median log-ER. Whiskers indicate the maximum length of 1.5 times the box length. Significance levels between all HR and LR fish are indicated on top of the plot (*t*-test on log-transformed values, ** = p < 0.01).

**Figure 7:**
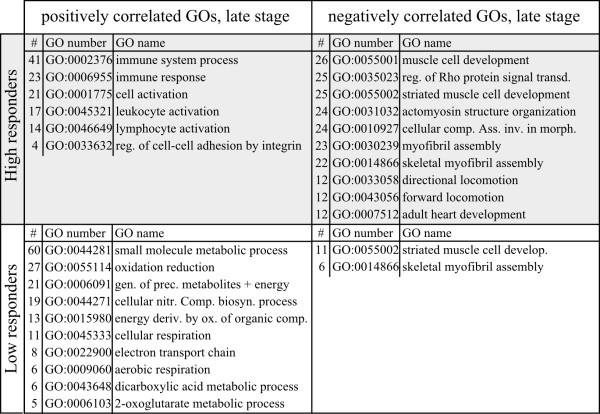
**Gene ontology classes correlated to viral load at late stage.** Gene ontology classes correlated to viral load in responder groups at the late stage of infection. Significantly enriched GO classes (FDR correction, p < 0.05) with positive or negative correlation to viral load (Pearson’s r > 0.6) in HR and LR groups at 8–10 wpi are shown. Column “#” shows the number of genes behind the respective GO term/class, and only the ten GOs with lowest p-values are shown (for completed data, see Additional file 4).

To substantiate these results, expression of four genes were evaluated by qPCR in an extended number of individuals per group (n = 9 HR, n = 10 LR, 8 wpi). Genes implicated in T cell responses were tested; *TNF decoy receptor* as marker of early pathology, *CD274* and *TNF-11b* as markers of late pathology, and *granzyme A* (*GzmA*) as a marker of cytotoxic T cells and the T cell response to CMS [10]. As shown in Figure 6, all genes had significantly higher relative expression ratios in HR versus LR (*t*-test on log_2_-transformed values, p < 0.01). In contrast to the other genes, *GzmA* was also upregulated in LR, however significantly lower than in HR. Taken together, induced transcription of these genes suggested an increased population and activity of T cells in infected hearts of HR compared to LR. Next we analysed differentially expressed GO categories between HR and LR that were positively or negatively correlated to the viral load at the late stage (Figure 7). As expected, positively correlated functional groups in HR included immune system process/immune response and more specifically, activation of lymphocytes and leukocytes. Negatively correlated groups included a large number of categories related to cardiac and skeletal muscle development (Figure 7 and Additional file 4). In LR fish, no immune-related GO categories showed correlation to viral load. In contrast, metabolic processes, generation of precursor metabolites/energy and oxidation reduction were positively correlated categories, while muscle cell development and skeletal myofibril assembly were negatively correlated.

### Microarray confirmation by qPCR

**Figure 8:**
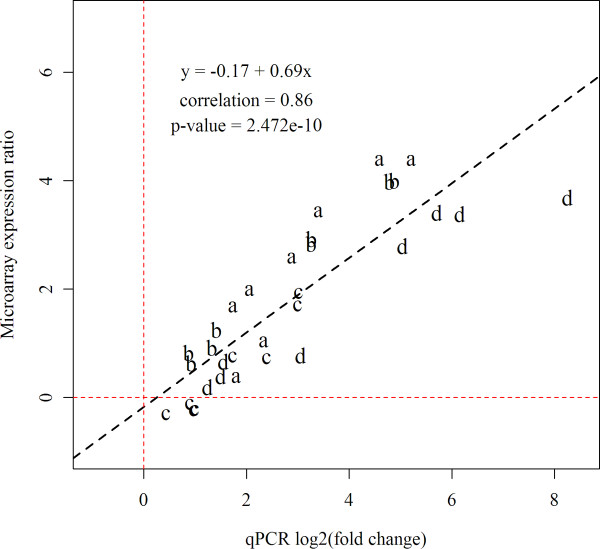
**Confirmation of microarray results by qPCR.** Comparison of gene expression results obtained with qPCR and microarrays. Log_2_-ER of four genes were compared (n = 8, plotted as letters; a = *radical s-adenosyl methionine domain-containing protein 2* (*rsad2*/*viperin*), b = *interferon-induced protein with tetratricopeptide repeats 5* (*ifit 5*), c = *retinoic acid inducible gene I* (*rigI*), d = *barrier to autointegration factor* (*baf*)). The dashed black line represents the regression function of the measured values. The regression model, correlation coefficient and p-value are shown in the graph.

To validate microarray data, log_2_-transformed expression levels of four early antiviral genes by qPCR were compared with the respective expression ratios from microarrays (Figure 8). Primers for qPCR (Additional file 5) and oligonucleotide probes on the microarray were based on the same cDNA sequences. Data from eight infected fish showed high correlation between the two methods.

## Discussion

In this study, we compared the host response in PMCV infected salmon with different outcomes of disease in order to characterize gene expression patterns and responses that could be associated with cardiac pathology versus recovery. Assessment of viral load and histopathology from bi-weekly samples of heart over a period of ten weeks suggested that fish exhibited different development of disease from six weeks post-infection. The so called high responders (HR) group developed severe pathology and increased infection level while the low responder (LR) group showed reduced viral load and retained a normal heart with absence of elevated pathology. This could be interpreted as different resistance to the disease since all fish were simultaneously infected using a standardized IP-injection with identical dose of virus. Previous analyses of fish from the same challenge trial also showed that all fish (n = 20) mounted a similar level of early antiviral response [10]. The early stage (2–4 wpi), was characterized by a rapid virus replication with no significant changes in myocardial tissue (histology score 0–1). However, results of enrichment analysis of GO classes and KEGG pathways showed repression of many functional groups related to cardiac and general muscle development, suggesting an onset of pathology at the molecular/cellular level. This could also be expected, given the strong antiviral response (at transcriptome level) in the cells at this stage, possibly leading to compromised cell growth. Both pathway analysis and gene expression profiling suggested that the early antiviral response involved genes of the interferon pathway and antigen presentation pathways via MHC, as was observed in our recent study [10]. Most of these genes were identified as virus-responsive with high correlation to expression of the salmon IFNa gene [12]. These are typical early responses to viruses and reflect the innate immune response followed by initiation of the adaptive immune response [24,25]. Thus, our results show that PMCV-infected cells successfully induced the transcription of many antiviral and IFN-dependent genes, but that this response had little or no direct effect on virus replication and outcome, since their magnitude of expression was negatively correlated with development of infection/disease and their expression remained induced within the whole challenge trial, except for the late stage in LR which had lowest viral loads. The same was observed in a study with viral haemorrhagic septicaemia virus infected rainbow trout, where genes of the interferon system were correlated to the viral load in affected tissue, however strong expression did not reflect a better protection against virus spread [26]. Nonetheless, these innate antiviral responses play a pivotal role in the activation of downstream adaptive immunity.

The mid stage of the disease was characterized by similar viral loads but different histopathology score in LR and HR. Pathway analysis showed a similar activation of most immune categories except for the LR-specific induction of NK cell-mediated cytotoxicity and NLR signaling pathways. These pathways were not induced at the early stage, but were later activated also in HR (late stage, 8–10 wpi). Despite a limited functional understanding of these responses in fish, they have important roles in the border between innate and adaptive immunity [27]. The earlier activation of NLR signaling (LR) in addition to the activation of Toll-like receptor pathway (LR and HR), may indicate a broader repertoire of virus-associated molecular pattern recognition in LR. For the analysis of predictor genes, our main hypotheses were that genes with stronger expression in HR than in LR represent predictors of early pathology, and genes with stronger expression in LR than in HR might represent predictors of protection/recovery. The predicted functional role of the majority of genes with significantly higher expression in HR (Figure 4) were interesting in view of the development of pathology in the subsequent late stage, which in our previous study was characterized by an increased inflammation dominated by influx of T cells [10]. In mammals, these genes are involved in cell death mechanisms and have been implicated in the control of lymphocyte regulation and survival. Induced transcription of these genes at mid stage of CMS in HR was likely a result of the initial rise in lymphocyte infiltration to the infected tissue as reflected from histopathology (this was the first stage with score 2, representing pathology above normal background levels, score 0/1). Since predictor genes were identified based on expression differences between HR versus LR, potential genes with equal expression in HR/LR at this stage were not taken into account. In this respect it is necessary to mention that many genes related to the T cell response in CMS [10] were upregulated already at the mid stage (see Figure 5 and Additional file 3), however differences between HR and LR appeared later. Thus, activation of lymphocytic and inflammatory responses occurred in both responder groups, but different abilities to control or regulate these responses might explain different outcomes in the subsequent stage. The importance of these responses in immunity versus immunopathology is intensively studied in human infections [28], and should deserve more attention in relation to viral infections in fish.

The recent studies on CMS showed a high correlation between histopathology score and viral load implying that the cytopathic effect of infection was a major determinant of the myocardial changes [9,10]. Results of this study further show that the level of pathology and infection is correlated to the activation of lymphocytes and leukocytes and particularly expression of genes associated with the T cell response at the late stage in HR. This was also expected, assuming that this response reflects the migration of virus-specific lymphocytes to the infected tissue. Infiltration of leukocytes/lymphocytes did not occur in LR fish according to histopathology results. This was accompanied by a completely absent expression of a large number of genes mainly related to T and B cell responses in the late stage, which was the most significant difference between HR and LR fish identified in this study. The lack of transcription of many genes involved in the regulation of T cell effector function and cell death/survival was of particular interest. Control of these processes is fundamental for regulation of the T cell response and for maintaining homeostasis in the immune system after it has expanded to combat infections [28,29]. As already discussed, many of these genes were also expressed at the mid stage of HR but not in LR. Elevated gene expression levels of *granzyme A* (*gzmA*) in LR fish showed that cytotoxic cells were also present in the heart of these fish. GzmA is a serine protease and important inducer of antiviral and apoptotic pathways in infected cells, produced by cytotoxic T cells and NK cells [30]. This suggests that adaptive cellular immune responses occurred in LR fish as well, however on a lower level compared to HR fish. Hence, both different shaping and a lower magnitude of immune responses could explain the different outcome of these groups. In contrast to HR, genes involved in energy metabolism and other catabolic/metabolic processes were induced in LR. In addition, the GO enrichment analysis showed that the same processes were correlated to the decline in viral load in LR. Thus, in contrast to HR, these fish seemed to cope with the infection by immune responses in the preceding stages and/or by a different composition or regulation of the late response, and managed to activate cardiac energy metabolism for recovery and regeneration of infected tissue in the late stage.

## Conclusion

**Figure 9:**
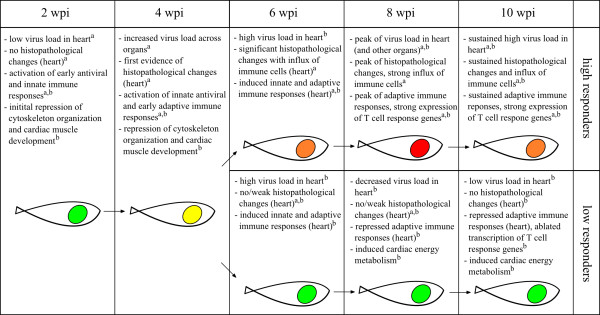
**Summary of pathogenesis in responder groups.** Summary of host-virus responses in high and low responder fish based on findings from a previous CMS study [10] and the present work, as indicated with superscript letters a and b, respectively. Colored circles in fish illustrate histopathology (atrium score level), analogous to Figure 1.

We have compared the host response and pathogenesis in PMCV infected salmon with different outcome of disease, and described gene expression patterns and predictors and associated functional pathways underlying these differences. The main findings are summarized in Figure 9, suggesting that very different composition and regulation of adaptive cellular immunity in the late stage of infection was the most prominent feature associated with pathology versus recovery. In our previous CMS study focusing on the most severely diseased fish, we also concluded that the same responses were possibly associated with an observed viral clearance and reduced pathology later on (10–12 weeks post infection) [10]. Consequently, while having an important role in the clearance of virus in infected heart tissue, virus-specific immune cells such as T and B cells may at the same time lead to increased tissue damage and burden for the host. Whether the stronger immune responses observed in HR was reflecting an immunopathology could not be concluded based upon the present data. However, the importance of understanding the balance between immunity and immunopathology is highly acknowledged in human disease [28], and should be subject to further study in viral diseases of Atlantic salmon. The basis for the positive outcome of LR fish is also not known. A recent field study with family group indicated a genetic basis for survival against CMS (N. Santi, personal communication). Fish used in the present study were from a commercial standard Norwegian strain with mixed genetic background. Thus, it could not be ruled out that the observed differences in disease response were influenced by genetic background. Effects of genetic background on disease development and host responses in controlled infection trials will be subject to future research.

## Methods

### Experimental infection and sampling

The biological material for this work was taken from a challenge trial previously described in [10]. In this trial, 120 fish were kept in four separated tanks; one infected and one control group in duplicates. The fish had an average weight of 50 gram at start. Each fish of the infected group received an IP-injection dose (0.2 ml) of an inoculate originating from a passaged cell culture developing cytopathogenic effects after infection with filtered heart homogenate derived from several adult Atlantic salmon with clinical CMS. Control groups received the same dose of conditioned cell culture medium. Tissue samples were collected 2, 4, 6, 8 and 10 wpi. At each sampling date, 15 fish from each of the four tanks were sedated (as described above) and euthanized by decapitation. For this study standardised samples from heart were snap-frozen in liquid nitrogen for RNA and fixed in formalin (10% neutral phosphate-buffered) for histology.

### Histopathology

Scoring of heart lesions by histopathological examination was taken from a previous study as described [10]. In brief, score 0 and 1 was considered normal, with no histopathological findings (score 0), or a single or few focal lesions (score 1). Score 2 represented several distinct lesions and increased mononuclear infiltration. Score 3 represented multifocal to confluent lesions in > 50% of tissue and moderate to severe leukocyte infiltration. Histopathology of individual fish shown in Figure 1 is based on histoscores from atrium.

### RNA extraction

Sampled hearts for microarray hybridization were stored at −80°C prior to RNA extraction. Standardised tissue sections of 10 mg (equal mix of ventricle and atrium) were prepared under sterile/RNase-free conditions and transferred directly to 1 ml chilled TRIzol (Invitrogen, Carlsbad, CA, USA) in 2 ml tubes with screw caps (Precellys®24, Bertin Technologies, Orléans, France). Two steel beads (diameter: 2 mm) were added to each tube and the tissue was homogenized in a Precellys®24 homogenizer for two times 25 sec at 5000 rounds per minute with a break of 5 sec between the rounds. RNA was extracted from the homogenized tissues using PureLink RNA Mini kits according to the protocol for TRIzol-homogenised samples (Invitrogen). The concentration of extracted total RNA was measured with a NanoDrop 1000 Spectrometer (Thermo Scientific, Waltham, MA, USA). The integrity of total RNA was estimated, using an Agilent 2100 Bioanalyzer with RNA Nano kits (Agilent Technologies, Santa Clara, CA, USA). Only samples with RNA integrity number (RIN) of 8 or higher were accepted.

### Quantitative real-time RT-PCR

Experiments were conducted according to the MIQE guidelines [31]. Synthesis of cDNA was performed on 0.2 μg DNAse-treated total RNA (Turbo DNA-free^TM^, Ambion, Austin, TX, USA) using the TaqMan® Gold Reverse Transcription kit (Applied Biosystems, Foster City, CA, USA) in 25 μl reactions with random hexamer priming according to manufacturer’s protocol. Complementary DNA was stored undiluted at −80°C in aliquots to avoid repeated freeze-thawing. To avoid risk for presence of residual DNA contamination, control reactions without RT was tested and qPCR primers were possibly designed to span introns. Oligonucleotide primers for genes of Atlantic salmon were designed with the program eprimer3 from the EMBOSS program package (version 5.0.0, http://emboss.sourceforge.net/). Amplicon size was set to 80–200 and melting temperature to 59–61°C. Primers were purchased from Invitrogen (Additional file 5). *In silico* analysis of gene targets was performed using a customised program for BLAST and sequence alignments [12]. PCR amplicon size and specificity were confirmed by gel electrophoresis and melting curve analysis (Tm calling; LightCycler® 480, Roche Diagnostics, Mannheim, Germany). QPCR was conducted in duplicate reactions as described [10]. Cycle threshold (C_T_) values were calculated using the second derivative method. Duplicate measurements that differed more than 0.5 C_T_ values were removed and reanalysed. For relative quantification, the mean of duplicates was used. Relative expression ratios of test samples versus the average of the controls were calculated according to the Pfaffl method [32]. Elongation factor 1α (GenBank ID: BT072490.1) was used as reference gene [33], and was found to be stably transcribed in control and test samples according to the BestKeeper software [34]. The efficiency of the PCR reactions was estimated for all primer pairs by six times 1:5 dilution series of a cDNA mix of all used samples. The efficiency values were estimated by using the LightCycler® 480 Software (version 1.5.0.39).

### Viral load

Relative quantification of PMCV was assessed by qPCR as described [10]. In brief, total RNA (62.5 ng per sample, prepared as described above) from infected fish (heart; weeks 0, 2, 4, 6, 8, 10, n = 5–8 per time point) were subject to cDNA synthesis (SuperScript® III Platinum® Two-Step qRT-PCR Kit with SYBR® Green, Invitrogen) and qPCR (2X Platinum® SYBR® Green qPCR SuperMix-UDG, Invitrogen) in triplicate reactions. Melting curve analysis was performed to confirm expected amplicon. Viral load was expressed as the relative copy number with non-infected controls (0 wpi) set to 1 and calculated by the formula 2^(C_T(0wpi median)_- C_T(sample)_).

### Design of microarray experiments

An overview of microarray hybridizations is shown in Additional file 1. The salmonid oligonucleotide microarray (SIQ2.0, NCBI GEO platform GPL10679) was used [12], consisting of 21 K features printed in duplicates on 4 x 44 K chips from Agilent Technologies. Two-color hybridizations in a reference design were used, where test samples labelled with Cy5 dye and reference samples labelled with Cy3 dye were competitively hybridized per array. As reference sample, pools of equimolar amounts of RNA from heart tissue from 6–8 control fish per time point were used (only fish with histopathology score 0 were included). The examined time points were 2, 4, 6, 8 and 10 wpi. For each time point, 5–8 individuals from both test (high and low responders) and control groups were hybridized against the reference sample, giving a total number of 65 arrays with 32 control (sham-injected) individuals and 33 infected (11 from the early stage, plus 11 HR and 11 LR from mid and late stages). HR and LR fish (mid and late stage) were identified based on histopathology score and infection level (viral load). From each time point representing these stages, 4 HR and LR fish were selected for microarrays except for 6 wpi, where one LR fish were excluded due to low viral load. From the early stage (2 and 4 wpi, before development of significant histopathology) all fish had equal viral load as previously identified [10], and 5–6 fish per each time point were randomly selected for microarrays.

### Microarray hybridization and data analysis

Unless specified otherwise, all reagents and equipment used for microarray analyses were purchased from Agilent Technologies and used according to manufacturer’s protocol. In brief, RNA labelling and amplification was performed with Low Input Quick Amp Labeling Kits, Two-Color and RNA Spike-In Kits, Two-Color for 4 x 44 K microarrays, using 200 ng of total RNA per reaction. For fragmentation of the labelled RNA, Gene Expression Hybridization Kits were used. Labelled RNA was hybridized for 17 hours (hybridization oven, Agilent) at 65°C and rotation speed of 10 rounds per minute. Arrays were washed for one minute with Gene Expression Wash Buffer I at room temperature, and one minute with Gene Expression Wash Buffer II at 37°C. Slides were scanned immediately after washing using a GenePix Personal 4100A scanner (Molecular Devices, Sunnyvale, CA, USA) at 5 μm. The laser power was manually adjusted to ensure an overall intensity ratio close to unity between Cy3 and Cy5 channels and with minimal saturation of features. The GenePix Pro software (version 6.1) was used for spot-grid alignment, feature extraction of fluorescence intensity values and assessment of spot quality.

Subsequent data processing and analyses were performed using the STARS platform [12]. Values of replicate spots passing quality control were averaged and Lowess normalization of log_2_-expression ratios (ER) was performed. Initial quality filtering was based on mean spot intensity and number of informative spots, resulting in 11,913 passed features. Outliers among control fish identified by cluster analysis (uncentered correlation, complete linkage using “Cluster 3.0” [35]) were removed. Significant differences between gene expression of infected and control fish were calculated by *t*-tests for each time point and group (HR and LR). The median values of respective control fish were subtracted from the individual values of the infected fish, and median log_2_-ER for each gene were calculated per group and time point. The final list of differentially expressed genes (DEG) was selected by filtering for previously mentioned *t*-tests (p < 0.05, at least one time point or group) and log_2_-ER > |0.7| (in at least one time point or group). Corrections for false discovery rate were not employed as previous microarray studies with Atlantic salmon have demonstrated them to be overly conservative [36,37]. Data was submitted to GEO (accession number GSE36860).

For identification of marker/predictor genes in HR and LR, correlation analysis was performed to search for (i) genes associated with the early antiviral response (strong induction at the early stage, moderate induction at the mid stage and late stage HR and no induction at late stage LR), (ii) genes with expression changes in mid and late stage HR and LR (iii) genes that were induced at only one stage and (iv) genes whose expression profiles correlated with virus loads. Equal thresholds were established for all correlations (Pearson’s r > 0.6).

Enrichment of GO classes and KEGG pathways among DEG were assessed with STARS (p < 0.05, Yates’ corrected chi square); terms represented with less than five genes were not taken into consideration. In addition, BLAST2GO [38] with an E-value cut-off for the BLAST searches of 10^-20^ was used for annotations and BiNGO plugin (Version 2.44, [39]) for Cytoscape (http://www.cytoscape.org/, version 2.8.0) for GO enrichment analyses. Only GO terms with the category “biological process” were considered and threshold of significance of the corrected p-value was < 0.05 (Benjamin & Hochberg false discovery rate correction). Multiple hits of identical probes were reduced to the GO term with highest occurrence (for complete lists, see Additional file 2). Part of plots and statistical calculations were made with the R software package (version 2.13.0, http://www.cran.r-project.org/*)*.

## Competing interests

The authors declare that they have no competing interests.

## Authors’ contributions

GT, SMJ and AK drafted the manuscript and conducted gene expression analysis. HT and SMJ planned the experimental design. MR and PN analysed viral load. SA developed the software for processing of microarray data and performed parts of the statistical analyses. All authors read and approved the final manuscript.

## Supplementary Material

Additional file 1 **Experimental outline.** Overview of experimental groups and number of biological replicates used for the different analyses. Click here for file

Additional file 2 **Primary data for Figure**2**.** Complete data from the enrichment analysis of GO classes and KEGG pathways. Click here for file

Additional file 3 **Primary data for gene markers and predictors.** Complete data used for the heat maps in Figures 3, 4 and 5. Data are median log_2_-ER for differentially expressed genes (DEG, *t*-test vs controls, p < 0.05) showing correlated expression (Pearson r > 0.6, correlation matrix in last sheet) to the different stages of infection in high and low responders (mid and late stages). Grades levels of red and green indicate respectively up- and downregulation. Click here for file

Additional file 4 **Primary data for Figure**7**.** Complete data from the analysis of GO categories between HR and LR that were positively or negatively correlated to the viral load at the late disease stage. Click here for file

Additional file 5 Real-time qPCR primers used in the study. Click here for file
